# Physical therapy in peripheral facial paralysis: retrospective study

**DOI:** 10.1016/S1808-8694(15)31131-9

**Published:** 2015-10-20

**Authors:** Márcia Regina Garanhani, Jefferson Cardoso Rosa, Alessandra de Mello Guides Capelli, Mara Claudia Ribeiro

**Affiliations:** 1 Master. Physical Therapist. Professor at the State University of Londrina; 2 PhD. Physical Therapist, Professor at the State University of Londrina; 3 Specialist. Physical Therapist; 4 Specialist. Physical Therapist

**Keywords:** exercises, bell's palsy, facial paralysis, physical therapy techniques

## Abstract

**Summary:**

Peripheral facial paralysis requires specialized treatment. Physical therapy aims at reestablishing facial movements. The aim of this study was to describe and to analyze physical therapy results for individuals with peripheral facial paralysis.

**Study design:**

Retrospective study. **Method**: A retrospective study was carried out at the University Hospital, authorized by the Statistics and Medical File Services, from 1999 to 2003. Data are presented in descriptive form with mean and median values for numeric variables and frequency for categorical variables.

**Results:**

Twenty-three files were analyzed during four years. Females predominated and the average age was of 32.3 years (SD±16.5); 14 idiopathic and five trauma cases; 12 with total motor deficit and 11 with partial motor deficit; in the 12 cases that underwent final evaluation, seven had partial and five had total recovery. The physical therapy program used was kinesiotherapy and patient education.

**Conclusion:**

In this study, individuals were similar to individuals in other populations. They were treated with kinesiotherapy, as suggested by the scientific literature and recovered.

## INTRODUCTION

Peripheral facial paralysis occurs from nervous input interruption at any of the facial nerve segments^1^,^2^. This may result in complete or partial paralysis of the facial muscles and may be associated to: tasting, salivation and tearing disorders, hyperacusia and hyposthesia of the external auditory canal2-4. In about 50% of the population affected by peripheral facial paralysis, the etiology is unknown. The first and foremost incidence is agnogenic, or Bell's palsy, the second is trauma, among others^2^,^5^. High blood pressure, diabetes mellitus, viruses, pregnancy and breastfeeding are deemed associated conditions^2^,^3^,^6^,^7^.

The degree of facial nerve recovery depends on patient's age, lesion type, nerve nurturing, neuromuscular involvement and therapy installed^2^,^7^,^8^. Facial nerve injury recovery may take weeks or up to four years^1^,^3^,^4^,^7^,^8^. Peripheral facial paralysis requires medical, physiotherapeutic and speech and hearing therapeutic approaches. Physical therapy is paramount, with the main goal of reestablishing muscle trophism, function and strength^7^, ^8^, ^9^. Approaches suggested in the literature are: Kinesiotherapy, massage and electro-thermotherapy, confirmed by random clinical assay and systematic review^7^,^8^,^10^,^11^.

The goal of the present investigation is to describe and retrospectively analyze physiotherapy results for individuals with peripheral facial paralysis.

## METHOD

We carried out a retrospective study on the charts of patients from January of 1999 to June of 2003. This medical chart survey was authorized by the Statistical and Medical Service, after being approved by the Research Ethics Committee, # 231/05. We found 35 charts from individuals seen at the physical therapy ward with diagnosis of peripheral facial paralysis. Of these, nine were excluded for not having a medical diagnosis, and three for not having any physical therapy record.

We analyzed 23 charts as to age, gender, side affected, initial and final motor compromise, etiology, time span between diagnosis and physical therapy beginning, treatment time, number of sessions and resources utilized.

The data collected are presented in a descriptive fashion, using average (Standard Deviation) and median (quartiles) for numeric variables and frequency (absolute and relative) for categorical variables.

## RESULTS

There was a predominance of females, 14 cases (60.9%). Average age was of 32.3 years (SD 16.5). There were 22 unilateral facial paralysis (95.7%), and 12 of those (52.2%) had their right side involved.

As to the facial paralysis etiology, we found 14 cases (60.9%) of agnogenic cause, five (21.7%), traumatic, three (13%) because of tumor and one case (4.3%), inflammatory. Of the individuals with agnogenic etiology, seven (50.0%) had total motor involvement and seven (50%) had partial motor involvement; of the traumatic etiology cases, two (40%) presented partial motor compromise and three (60%) had total compromise; of the tumor cases, two (66.7%) had partial motor involvement and one (33.3%) had total motor compromise; and the only case of inflammatory etiology presented total motor compromise.

In the initial evaluation, motor compromise was total in 12 cases (52.2%) and 11 had partial compromise (47.8%). Of the 23 charts analyzed, only 12 had the final individual's assessment at the time of discharge. Such fact limited the analysis as to patient recovery after the physical therapy sessions. In these 12 cases analyzed we saw that seven (58%) had partial recovery (paresis) and five (41.7%) had total recovery. In analyzing initial and final compromise, all patients with total motor involvement evolved for either a normal or partial status. Nonetheless, only a few cases of paresis evolved to a normal status.

Time span between medical diagnosis and the onset of physical therapy was from two to 136 days, with a median of 15 days (seven and 22 days - 1st and 3rd quartiles). As to the duration of physical therapy, the patients remained within the 12 week median (eight and 21.1 - 1st and 3rd quartiles) in treatment with individual 45 minute sessions.

The most used therapeutic resources were: sensorial stimulation, propioceptive neuromuscular facilitation exercises and guidance (100%); massage (95.7%); stretching exercises (69.6%); pompage (26.1%) and electrotherapeutic (26.1%). Electrotherapy was carried out by means of a transcutaneous electrical stimulation, aiming at pain relief.

## DISCUSSION

Peripheral facial paralysis causes and frequencies found in this study are in agreement with those found in the Brazilian literature^3^,^8^. According to Nakamura et al.^12^, the frequency of agnogenic facial paralysis varies from 62% to 93% of the cases. However, a Colombian study showed a higher rate of traumatic facial paralysis when compared to Bell's palsy13. According to Vasconcelos^2^, the second highest incidence of peripheral facial paralysis is traumatic in origin, and it may occur as a consequence to fractures in facial bones, such as it happens in head injuries.

Approximately one third of the patients end up with significant cosmetic or functional sequelae such as: oral dysfunction, muscle contractures, nasal obstruction, dysgeusia, dysesthesia, syncinesis and hemifacial spasms^3^. Often times, the complete lesion recovery is prevented because of syncinesis.^4^,^7^,^8^ Syncinesis may be related to physical therapy being applied without supervision and electrostimulation14. This is a sequela related to facial nuclear hyperexcitability, or the aberrant regeneration of nervous fibers1. The present investigation showed that, among the 12 cases with final evaluation, seven presented with motor sequelae and only one had syncinesis.

When we compare etiology to motor recovery (n=12), we noticed total recoveries in the cases of agnogenic etiology (60%) and traumatic (40%). In a study involving 140 patients with agnogenic facial paralysis, Wolf^15^ reported a satisfactory recovery in 82.1% of the patients.

Although it is common to have a worsening of symptoms in the first 48 hours, most patients evolved to recovery within some weeks. Nonetheless, when there is evidence of denervation after 10 days, there may be a delay in recovery onset (three months in average)^4^,^7^. Ribeiro^8^ stated that the average time for facial nerve recovery may be from 15 days to four years. Cohen^16^ observed that, in 95 pregnant women, complete recovery of Bell's palsy happened in 56 patients (58.9%) within four months or less. In a study involving 36 patients with peripheral facial paralysis and using Kinesiotherapy, partial recovery was seen in 83.3% of the patients after 15 days, and total recovery in 63.8%, after 30 days of physiotherapy^17^. The participants in this study remained in the 12 week treatment median, and this may mean early discharge or treatment abandonment; three remained for more than one year and only one was followed up for four years.

Studies advocate stimulation with quick massage and facial movement exercises in order to improve symmetry. In an international meeting held two decades ago, it had been already advocated the following up of peripheral facial paralysis patients with exercises, massage, electrotherapy and biofeedback^7^,^8^,^12^,^17^,^18^. Cronin & Steenerson^14^ propose biofeedback by surface electromyography. Results show an improvement in facial symmetry and in syncinesis in 24 patients.

Neuromuscular training for facial movement exercises are used in order to improve facial symmetry^9^,^4^. The main physical therapy resources employed in the patients of the present study were: kinesiotherapy, massotherapy, cryotherapy and electrotherapy. Kinesiotherapy with neuromuscular facilitation and sensory stimulation were used in all interventions. The results attained in facial training may be explained by the theory of nervous system plasticity^19^.

Of the 23 charts analyzed initially, only three patients received electrotherapy associated to kinesiotherapy for pain control, and not neuromuscular electrostimulation. The neuromuscular electrostimulation program is able to partially revert motor deficit and the sequelae of peripheral facial paralysis, when combined with a specific exercises program. A narrative and systematic review of the literature show benefits of using classical electrostimulation and biofeedback; and concluded that more studies are necessary in order to reach the specific parameters of utilization and evaluation of these benefits, in order to truly prove their effectiveness^11^,^18^. A comparative study involving 49 patients with Bell's palsy showed a fast and complete recovery in 77 patients treated by neuromuscular electrostimulation, compared to 72 patients treated only with prednisone^20^. Nonetheless, electrotherapy may be responsible for an increase in tetany and hypertonia that cause syncinesis. This treatment modality has been abandoned in favor of analytical muscular work and muscle stretching exercieses^9^, ^10^, ^11^, ^12^, ^13^, ^14^.

All the patients in the present study were instructed as to the care they should take in domestic activities. Basically, such education aims at providing patients with information, prevent symptom recurrence and to cause a change in the behavior of these individuals^7^. In this sense, it significantly favors changes in health care, since it makes it easier for the affected individual to accept responsibility for his/her treatment.^21^.

## LIMITATIONS AND SUGGESTIONS

The records of the cases hereby presented are incomplete and not standardized. It is fundamental to insert in the physical therapy department protocols that may come to facilitate the recording of data from individuals with facial paralysis referred for treatment, such as: medical treatment employed, initial and final motor evaluation, resources selected, treatment report, thus allowing for more analytical studies.^22^

The many resources used in the physical therapy of individuals with facial paralysis cause neuromotor recoveries. However, we need studies that show the true effectiveness of these resources, with randomized clinical trials, aiming at enhancing the clinical decisions of physical therapists.

## CONCLUSION

The present investigation showed that the characteristics of individuals with facial paralysis seen at the physical therapy ward are similar to those from other populations. They had an improvement in their initial symptoms after treatment employing kinesiotherapy, as suggested by clinical practice and the scientific literature.
